# Report on the Memoirs of Drs. Miquel, of Ambrise, and Stern, of La Haye, on a Mode of Tamponing the Genital Passage in Cases of Uterine Hemorrhage in Pregnant Females

**Published:** 1849-05

**Authors:** James Bryan

**Affiliations:** Professor of Surgery in Geneva Medical College, President of the College of Physicians and Surgeons of Philadelphia


ART. III.—*Report on the Memoirs of Drs. Miquel, of Ambrise, and Stern, of La Haye, on a Mode of Tamponing the Genital Passage in cases of Uterine Hemorrhage in Pregnant Females.* Commissioners,—FLOURENS and ADRAL, VELPEAU, Reporter. (Translated by JAMES BRYAN, M. D. Professor of Surgery in Geneva Medical College, President of the College of Physicians and Surgeons of Philadelphia.)

The Academy has confided to Messrs. Flourens, Andral, and myself, a work of Doctor Miquel, describing a new method of remedying the dangers of the implantation of the placenta on the neck of the uterus, during gestation. It has also submitted to us a dissertation of M. Stein on nearly the same subject. The object of these authors appearing to be about the same, and the means which each has contrived, very analagous, we have concluded to consider both in one report.

Gestation exposes certain females to a species of hemorrhage, the cause of which is found in the implantation of the placenta in the vicinity of the neck of the uterus. This hemorrhage, almost inevitable, because it is connected with the anatomical arrangement and physiological development of the organs, is so dangerous, that it not unfrequently causes the death of both mother and child, in spite of the most assiduous and patient attention. Since the commencement of the last century, the time when accoucheurs turned to it their special attention, many means of cure have been suggested; yet all practitioners have continued to sigh over the inefficiency of art, in this matter. It is this, which gives interest to the works of Miquel and Stein. Both these authors propose remedying this species of hemorrhage, by means of the bladder of an animal, introduced empty into the organs, and there distended by a fluid, water, for example, or air, in a manner to produce a large tampon.

Before proceeding farther, we should say, that the *bladder* employed in this manner, is not an absolutely new remedy. It was mentioned in the last century by several authors, Walbaum, Schlichting, Lenay, Basedone› and others. In our times by Rouget, Galbiati, and Verdier, among others. But it is a means which has not remained in practice: Miquel and Stein present it in another point of view, as well as under another form, and accompanied with arguments which their predecessors did not have.

Although the bladder has been proposed by both authors, the remedy is different in the hands of each. For this reason we will examine them separately.

*Report on the Process of Stein.*—Stein's object, was to establish a compression on the free portion of the uterus, in the upper part of the vagina, and also to oppose an obstacle to the passage of the blood, from the internal surface of the neck of the uterus; or from the external surface of the placenta. For this, he made use of means, thus arranged: He procured the bladder of a goat or sheep, prepared, and furnished with a solid ring; a metallic canula, open at both ends, widened like a funnel at the lower end. Armed with a lateral facet, screwed upon the upper part of the bladder, which was introduced, empty, to the upper part of the vagina. Having blown it full of air, or filled it with water, by means of a syringe, it only remained to close the facet, open up to this time, to complete the operation.

In this way, we have a body enlarged to any given size, which fills exactly the vagina, without fatiguing the parts so much as the other tampons usually employed. As the bladder filled with water may be, other things being equal, easily compressed, it fills without irritation all the inequalities of the parts; while the sides of the neck of the uterus, for example, depressing it centrally, are pressed all around by a species of soft circular pad, which fills the upper cavity of the vagina, and compresses that portion of the uterus, which is ordinarily in the vagina, at the time of labor.

The weight of the fœtus and its dependencies, will present an obstacle to the hemorrhage, in supporting itself upon the placenta from above downwards, while the apparatus of Stein resists from below upwards. The same may be said of the resistance offered by the parietes of the uterus, and their contraction, whether the labor has commenced, or the uterus be in its normal condition.

In order to understand the indications of this means, it must not be forgotten that the blood comes from the interior, from the vicinity of the orifice of the uterus, and that it runs from that part because the placenta is found in the lower part, and not above. Indeed, if the hemorrhage occurring from any other cause, came from some other part of the uterus, the means of which we speak, instead of being useful, might be very dangerous, preventing the blood from passing outwards, and retaining it in the interior, without preventing the hemorrhage from the veins of the mother. When hemorrhage occurs from the implanting of the placenta on the neck, however, tamponing the vagina, is very beneficial. By this means we close the orifice of the organ, and as the placenta is immediately above, it follows that the vessels are compressed, by the fœtus on one side, and the bladder on the other.

All obstetricians agree, at the present day, on this point. In this matter, then, Stein need fear no opposition from practitioners, while he does not add to the knowledge we already have. But the end which is proposed by his process, differs from those already admitted, in the following manner: To tampon the vagina, in females with hemorrhage, we introduce charpie, and increase the quantity either alone, or through a bag of fine muslin; sometimes instead of charpie, we use tow-carded flax, or pieces of old linen, or even a handkerchief. Some use a roll of cloth, or introduce it in pieces.

Such a tampon is certainly much better than nothing, for according to an old axiom in bad cases, it is better to use a doubtful remedy than nothing. In general, however, these tampons are insufficient; they become lumpy, of unequal consistence; they press too little or too much; now against the urinary bladder, now against the rectum; they press more against the neck, than against the neighboring parts of the uterus. They imbibe the blood, become loose, and are soon displaced; and thus act but very imperfectly in arresting the hemorrhage.

With the apparatus of Stein, the foreign body used as a tampon, is depressed on the surface very easily, and adapts itself to the inequalities of the parts; pressure is made against the uterine region, or the flowing blood, in an equal and continuous manner. We can, at pleasure, and without pain, augment or diminish the volume, or the density of the hemostatic bladder. As this bladder is impermeable, the blood does not penetrate it, and hence there will be no deception in its use, as in the tampons of linen.

We must not expect, however, that the dangers of hemorrhage, from the implantation of the placenta on the neck, will be thus easily overcome. If, instead of being in the vicinity of the neck of the uterus, the torn vessels are some distance beyond, the apparatus will not stop the hemorrhage any better than the others.

All that we can expect from it is, when tamponing is indicated, this method is the surest, the easiest, and the least troublesome that we have, and that, always, when the tamponing is to be applied only to the interior of the vagina. We will see, soon indeed, that Miquel introduces it, even to the interior of the uterus.

Stein closes his memoir, with a dissertation on the causes of the implantation of the placenta on the different parts of the cavity of the uterus, and the mechanism of the hemorrhages which are the result of this implantation. The reflections of the author, tend to establish these two facts, that the fixation of the placenta on one or other point of the uterus, and the appearance of hemorrhages, depend upon a species of anormal contractility of the fibres of the uterus. But what he says on this subject, being but the result of simple theoretic views, unsupported by experience or precise observation, does not appear to us worthy of occupying much of the time or attention of the Academy.

In closing, we would say, first, that the hæmostatic process proposed by Stein, is useful, and should be preferred to ordinary tamponing, in cases of hemorrhage from the implantation of the placenta on the neck of the uterus. Second, that the bladder indicated by this physician, and employed by others, has not been recommended, either under the same form, or with an object exactly the same. Third, that the entire work of Stein, is one worthy the esteem and approbation of the Academy.

It should be added, in conclusion, that this gentleman gives the credit of the invention of the proceeding which he extols, to Wellamberg, his master, a distinguished accoucheur of La Haye. The description has even been published for some twelve years, in the country journals, and he published this memoir, only to describe an improvement which he has made to the apparatus, and to obtain the opinion of the Academy, on his entire work.

The conclusions of this report were adopted.

---

*Report on the proceeding by Miquel.*—Struck, like other physicians, with the great dangers of hemorrhage, from implantation of the placenta on the neck; convinced by experience, of the uncertainty of the various known modes of tamponing; Miquel attempted to use the bladder, in a manner quite new. It is not, indeed, in the vagina, but in the interior of the uterus itself, that this physician establishes his system of compression.

The apparatus of Miquel is composed, first, of a pig's bladder, second, a metallic canula, from 18 to 20 centimeters long. Third, a double ribbon, to fasten the body of the bladder on the canula, and to fix at another part, the neck of the bladder to the back of the canula. Fourth, a baton with a blunt extremity, to sustain the summit of the animal pouch, while it is introduced; and five, a species of little baton, on which are fastened the two ties just mentioned.

To apply it, the female is placed in the ordinary position for artificial labors. Carried on the finger, or by the aid of a speculum, up to the neck of the uterus, the bladder must be introduced, either along side of (or across) the placenta, if it occupies the center of the orifice, or between the ovum and the parietes of the uterus; the mandrin is then withdrawn; air or water is then injected by means of a syringe, so as to distend fully the pouch thus placed upon the neck. The external opening of the canula is then carefully closed by a facet, if there be one, or by means of a cork. The ends of the ties which fasten the bladder, near the middle, to the canula, and of that which closes the external extremity, are then fixed on the little baton, mentioned above, to prevent all kind of slipping. These ties, and the stick which supports them, are made to produce traction from above, downwards, to compress all the internal surface of the summit of the uterus, better than the head of the fœtus can do it.

We thus understand the mechanism of the tampon apparatus, and the object aimed at, by Miquel. By the method of Stein, compression from below, upwards, does not meet with sufficient resistance from the ovum, to satisfy the practitioner. Limited by adhesion of the vagina, the action of the Holland Physician's tampon, is not sufficiently extensive to cover the whole surface from which the blood flows. Its action being established outside of the uterus, may not, perhaps, induce contractions; at the same time, it does not prevent a collection of blood in the uterus, or even in the coverings of the fœtus.

By the method of Miquel, on the other hand, all the indications are met, and several inconveniences avoided. Once in place, the pouch prepared by this physician may take almost any size or tension, at the will of the surgeon. In drawing downwards, we are sure of applying pressure, either directly, or through the medium of the placenta or the membranes, to the vascular orifices. This compression may extend to the fourth, or third of the uterine cavity, and will certainly extend beyond the limits of the bleeding disk, representing, to a certain extent, a second head of the child. The bladder thus placed and distended, has all its efficacy. We see, on the contrary, its action is augmented by the contractions of the uterus, under the influence of labor. It is thus evident, that this manner of tamponing is, at least, more efficient and more formidable, than that of Stein; more efficacious, because, well done, it inevitably arrests the hemorrhage, while the other method frequently fails; more formidable, because once in place, the internal tampon will excite, without doubt, uterine contractions, and premature labor, which will not necessarily follow the application of the vaginal tampon.

We must, then, in this matter, establish a distinction. Before the seventh month, while the viability of the fœtus is yet doubtful, if there be no urgent necessity, on the part of the mother, to finish labor at once, the plan of the physicians of La Hage merits, it appears to us, the preference. If, on the other hand, the dangers to the female are very great, or if gestation is so advanced that the expelled fœtus has a good chance of living, we should resort to the process of Miquel.

We may add, that this method is a little more complicated than the other; that the apparatus, to be introduced without danger to the mother or fœtus, requires skill and patience; but to men acquainted with the accident, these inconveniences are of little consequence.

Fully to appreciate the value of these resources, one must have witnessed the frightful position in which the accoucheur is placed, in these unhappy cases; one must picture a poor woman, otherwise well, arrived at the seventh month of gestation safely, and who after that, suffers fatally, inevitably, an unceasingly increasing hemorrhage; a hemorrhage which will often destroy both her and her infant, whatever is done, unless, by some mechanical means, the bleeding vessels are stopped. One must, in fine, imagine the painful alternative of penetrating the uterus to extract a fœtus, or allowing it to be expelled nearly dead, or in a condition to die, in a few hours; or to tamponise, to preserve the product of conception as long as possible, at the risk of compromising, more and more, the life of the mother, and see a young, and, until then, vigorous female, sink without help. It is for this reason, that Miquel, having suffered for more than thirty years, in an extensive practice in the vicinity of Tours and of Amboise, has proposed the apparatus which we have feebly sketched. The work of the author is the result of an extensive practice; in it, he presents cases which show the uselessness of the ordinary modes of tamponing, and others, which exhibit the efficacy of the plan proposed by himself. Persuaded that this method of tamponing will afford valuable assistance, and that it is, for this reason alone, proper to make it known by considerable publicity, we propose to the Academy to insert the work of Dr. Miquel among the *memoirs of learned foreigners.*

The conclusions of the report were adopted.

